# Epstein-Barr virus latency switch in human B-cells: a physico-chemical model

**DOI:** 10.1186/1752-0509-1-40

**Published:** 2007-08-31

**Authors:** Maria Werner, Ingemar Ernberg, JieZhi Zou, Jenny Almqvist, Erik Aurell

**Affiliations:** 1 Computational Biological Physics, School of Computer Science and Communication, Royal Institute of Technology, AlbaNova University Center, SE-106 91 Stockholm, Sweden; 2 Department of Microbiology, Tumor and Cell Biology, Karolinska Institute, SE-171 77 Stockholm, Sweden

## Abstract

**Background:**

The Epstein-Barr virus is widespread in all human populations and is strongly associated with human disease, ranging from infectious mononucleosis to cancer. In infected cells the virus can adopt several different latency programs, affecting the cells' behaviour. Experimental results indicate that a specific genetic switch between viral latency programs, reprograms human B-cells between proliferative and resting states. Each of these two latency programs makes use of a different viral promoter, Cp and Qp, respectively. The hypothesis tested in this study is that this genetic switch is controlled by both human and viral transcription factors; Oct-2 and EBNA-1. We build a physico-chemical model to investigate quantitatively the dynamical properties of the promoter regulation and experimentally examine protein level variations between the two latency programs.

**Results:**

Our experimental results display significant differences in EBNA-1 and Oct-2 levels between resting and proliferating programs. With the model we identify two stable latency programs, corresponding to a resting and proliferating cell. The two programs differ in robustness and transcriptional activity. The proliferating state is markedly more stable, with a very high transcriptional activity from its viral promoter. We predict the promoter activities to be mutually exclusive in the two different programs, and our relative promoter activities correlate well with experimental data. Transitions between programs can be induced, by affecting the protein levels of our transcription factors. Simulated time scales are in line with experimental results.

**Conclusion:**

We show that fundamental properties of the Epstein-Barr virus involvement in latent infection, with implications for tumor biology, can be modelled and understood mathematically. We conclude that EBNA-1 and Oct-2 regulation of Cp and Qp is sufficient to establish mutually exclusive expression patterns. Moreover, the modelled genetic control predict both mono- and bistable behavior and a considerable difference in transition dynamics, based on program stability and promoter activities. Both these phenomena we hope can be further investigated experimentally, to increase the understanding of this important switch. Our results also stress the importance of the little known regulation of human transcription factor Oct-2.

## Background

Epstein-Barr virus (EBV) primary infection usually occurs early in childhood until teens, and then persists through-out life as latent infection in a fraction of B-lymphocytes in more than 90% of adults. One adverse consequence of the infection is an increased tumour risk [1]. There are some 170.000 new EBV-positive tumours occurring annually in the global human population, of which half derive from the hematopoietic compartment and half from epithelial precursors. The tumour risk is thought to be intrinsic to the viral strategy for survival and spread. Indeed, the ability of the virus to transiently induce proliferation of latently infected B-lymphocytes results in an increased pool of infected cells. This induction of proliferation depends on the switch between viral latent programs in the cell, which can bring the cell from resting state, into active cell cycle and back to resting. If this switch gets out of balance, more proliferation leads to a higher load of virus-infected cells and hypothetically increases the risk for lymphoma development. The mechanisms controlling induction of proliferation are not understood. The most upstream event that can be identified until now is the switch between two viral promoters, the Q promoter (Qp) and the C promoter (Cp), which in turn determines expression of key regulatory viral proteins. Here we present an *in silico *study of this viral switch, involving one viral and one human protein. The aim is to investigate whether this simplified model can explain the availabel experimental data, as well as opening the field of EBV research to modeling.

The EBV genome is a 172 kbp long double-stranded DNA which during latent infections is maintained in the nucleus of the host cells as an extra-chromosomal circular episome. Twelve viral genes can be expressed in different combinations during latent viral infection, while the remaining 70 major open reading frames are expressed during the replicative, lytic cycle [2]. During latency EBV genes are expressed in four programs, denoted latency 0, I, II and III. In latency III all 12 latency genes are expressed, including six nuclear proteins, EBNA-1-6, three membrane proteins (LMP-1, LMP-2A and LMP-2B), BART and two small non-translated RNAs (EBER 1 & 2). In latency II, the viral genes for EBNA-1, the three membrane proteins and the EBERs are expressed while in latency state 0/I, only LMP2a and variably EBNA-1 are expressed [2,3]. B-lymphocytes in latency III are proliferating, driven by the viral gene products, while the remaining latency forms are in non-proliferating, resting cells. An essential viral protein is EBNA-1; responsible for viral replication, episome partitioning as well as functioning as regulator of gene transcription [4]. EBNA-1 is therefore expressed in all latent programs, but the mRNA transcripts results from different promoters; Cp in latency III and Qp in latency I [5-8]. It is believed that the two promoters are mutually exclusive due to different EBNA-1 levels between the latency programs [6,9].

The Cp transcripts produce all six EBNA-proteins from bicistronic mRNAs modulated by alternative splicing [10]. Its activity is directed by several regulatory sequences of which the enhancer 'family of repeats' (FR) region is the major regulatory site. FR consists of multiple EBNA-1 and octamer binding sites, in an alternating pattern [11], and EBNA-1 binding to FR is required for transcriptional activation [6,12,13]. The enhancement of Cp by EBNA-1 bound at FR follows a complicated pattern, where at least eight of the 20 present sites need to be occupied by EBNA-1 for full transcriptional activation [14,15]. The human transcription factors Oct-2 and Oct-1 in association with Bob.1, have been shown to activate Cp, while Oct-2 in association with members of the Groucho (Grg/TLE) – family of proteins acts as an inhibitor of Cp [11,16]. Oct-1 is however not the prime candidate for operating the switch since it is not B-cell specific, like Oct-2, and moreover there is little or no difference in Oct-1 levels between latency I and III cells (Almqvist J et al.: Repression of Epstein-Barr virus enhancer Family of Repeats mediated transcription by Oct and Grg/TLE transcriptional regulators, suggest an involvement in switching between latency states, submitted). Oct-2 levels, on the contrary, differ between latency I and III (as shown in this study), indicating that Oct-2 is the dynamic inhibitory protein, even though it requires to bind FR in complex with Grg/TLE to be inhibitory [16].

Between FR and the initiation site there are other regulatory sequences such as an EBNA-2-dependent enhancer [17,18], GRE, a glucocorticoid-responsive enhancer [19], and sites for Egr, Sp1 and NF-Y transcription factors [20]. Deletion analysis however indicated that the GRE is not necessary for transcription initiation, and the EBNA-2 dependent enhancer is not sufficient to activate Cp [21].

Moreover, Sp1 and NF-Y are two ubiquitously expressed proteins known to interact with each other [22], and NF-Y is suggested to play a role in basal transcriptional regulation [22,23], with binding sites in 30% of all eukaryotic promoters [24]. Taken together, these experimental data indicate that EBNA-1 is the major activator of the C promoter.

In contrast to Cp, Qp governs expression of a monocistronic EBNA-1 transcript and is thought to be a housekeeping promoter of latency I since it lacks TATA sequence upstream of the initiation site, like many cellular housekeeping genes [25]. Experimental studies of Qp regulation have revealed three types of transcription factors binding in the promoter region; EBNA-1, E2F and IRF. IRF factors have been shown to constituitively activate Qp, with the role of directing general transcription factors in the absence of a TATA box [9,26]. The information concerning E2F regulation is less consistent, since E2F has been shown to both activate and inhibit Qp [27,28]. There is however no doubt that Qp is negatively auto-regulated through binding of EBNA-1 to the two binding sites downstream of the initiation site [6,8,27,29], and that one occupied site is enough to block Qp activity [6].

Transcriptional control of genes is not trivial to model, especially not for eukaryotic systems with complex regulatory machinery. However, a main dynamic regulatory mechanism is the binding of inhibitory or stimulatory transcription factors to operator sites on the DNA. Assuming that the rates for binding and unbinding of transcription factors are much faster than the rates for open complex formation and elongation, these bindings are in homeostatic equilibrium with the instantaneous transcription factor concentrations [30,31]. This is the basis for modelling gene transcription probabilities with thermodynamic models, as has successfully been used to describe genetic switches in coliphage systems [30-33].

**Figure 1:**
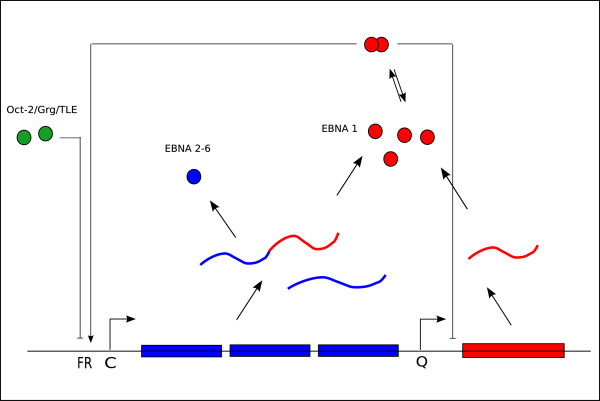
**The genetic switch**. This illustration describes the regulatory mechanisms of the modeled switch between the C promoter (Cp) and the Q promoter (Qp) in Epstein-Barr virus. Transcripts from Cp are spliced into bicistronic mRNAs coding either for EBNA-1 (red) or one of the other EBNA proteins (blue), while transcripts from Qp only codes for EBNA-1. EBNA-1 itself acts as a negative regulator of Qp, binding downstream of the transcription start, and positively regulates transcription from Cp through binding upstream to the Family of Repeats sequence (FR). FR bind also Oct-2 molecules (green), in complex with Grg/TLE, acting as inhibitory regulators.

In this paper we construct a physico-chemical model of the switch between EBV latency I and latency III. The model is based on the hypothesis that promoter control by EBNA-1 and Oct-2 is sufficient to switch between Cp and Qp usage in the two latency programs. Even though this is a simplification, the experimental data summarized above point to EBNA-1 as the major regulatory factor in controlling both promoter activities, and to Oct-2 in complex with Grg/TLE as a candidate for inhibitory control of Cp. In our model, Cp is thus switched on by EBNA-1 and switched off by Oct-2+Grg/TLE, while Qp is switched off by EBNA-1 and is otherwise on. Figure 1 gives a schematic overview of the model.

While this system belongs to the same general class of systems studied previously, a distinguishing feature of our system is the relatively large number of number of binding sites, and the complicated observed dependence of activation on the number of EBNA-1 bound. This theoretical approach to understand EBV-induced cell proliferation is to be seen as an extension of experimental studies and a first step towards a system level description of EBV infections.

## Results

**Figure 2:**
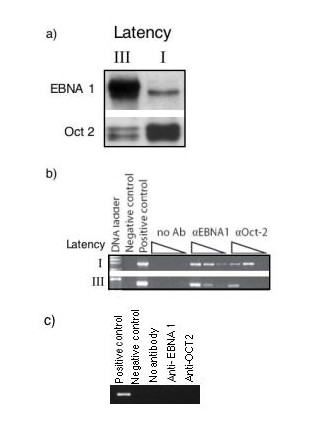
**Protein levels and DNA binding**. a) Western blot showing EBNA-1 and Oct-2 in latency I type cell Rael (I) and latency III type cell CBMI Ral-STO (III). Monoclonal antibodies were used to detect the proteins as described in Methods. b) Chromatin immunoprecipitation(CHiP)-assay of EBNA-1 and Oct-2 probed by PCR over the left hand of FR. The antibody-protein cross-linked and precipitated DNA was probed undiluted, diluted 1:10 and 1:100. The sources of the protein-FR complexes were the same cell lines as in a). Controls were immunpreciptation leaving out the first specific antibody, a cloned, purfied piece of FR (positive control) and PCR with no DNA (negative control) c) Control of non-specific binding of antibodies to DNA. DNA from latency III cell lysate was immunopreciptated with anti-Oct2 or anti-EBNA1, with negative and positive controls. Results show no significant background binding of the antibody.

The two states latency I and latency III can be studied in human EBV-positive B-cell lines either derived from tumors or transformed *in vitro *by the virus. Cells in latency I without exception show lower levels of EBNA-1 and higher levels of Oct-2 compared cells in latency III ([11]; Almqvist J et al.: Repression of Epstein-Barr virus enhancer Family of Repeats mediated transcription by Oct and Grg/TLE transcriptional regulators, suggest an involvement in switching between latency programs, submitted). An example of a qualitative comparison of EBNA-1 and Oct-2, as detected in Western blot in the two types of cells, is shown in Figure 2a. We have estimated the quantitative differences between the intensities of the bands in Figure 2a, and in several different pairs of latency I and III cells (data not shown), using the ImageJ densitometry program (NIH). The EBNA-1 signal was between 2.8–4.7 times higher in latency III compared to latency I cells, while Oct-2 was 2.1–3.3 times higher in latency I cells compared to latency III. In vivo binding of both EBNA-1 and Oct-2 to FR in these two cell types can be demonstrated by a CHIP-assay (Figure 2b).

### Latency states in the model

**Figure 3:**
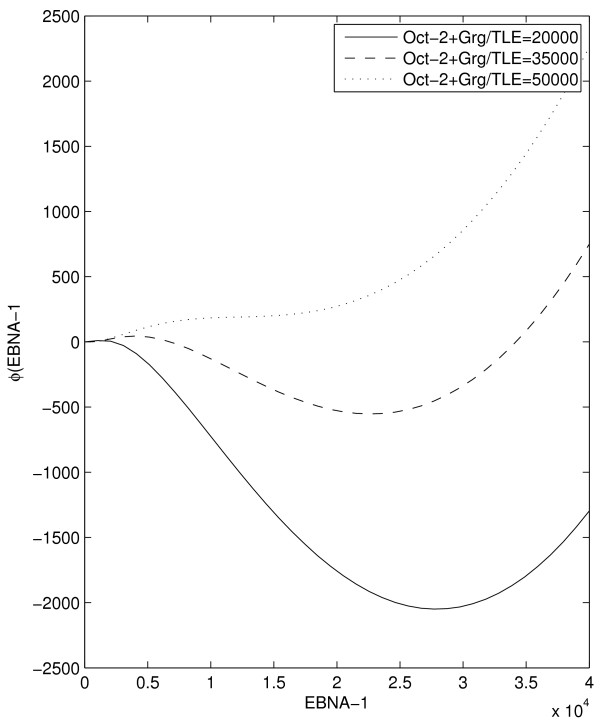
**Potential landscape**. The potential landscape of the EBV kinetic system for three different levels of Oct-2+Grg/TLE. The two local minima in the potential landscape corresponds to latency I, at low EBNA-1 levels, and latency III, at high EBNA-1 levels. Increasing the Oct-2 levels in the system shift the latency III minima towards latency I and eventually it disappear completely, leaving the system in a monostable latency I state.

Our model displays regions of mono-stability, with either latency I or III as the only stable state, and a region of bi-stability. For low Oct-2+Grg/TLE levels, only latency III is stable, while, for higher levels, there is a bistable region. This eventually gives way to the monostable latency I, at even higher Oct-2+Grg/TLE levels. The net effects of production from the two promoters together with dilution and decay can conveniently be visualized by a production potential. The negative derivative of this potential, with respect to the number of EBNA-1, correspond to the net production rate of EBNA-1. Figure 3 displays this production potential for three different levels of Oct-2+Grg/TLE. At intermediate Oct-2+Grg/TLE levels, the system is bistable, i.e. the production potential has two local minima (dashed line). Increasing the Oct-2+Grg/TLE level eventually eliminates latency III (dotted line), while decreasing Oct-2+Grg/TLE level eliminates latency I (solid line).

In our model, the level of EBNA-1 in the two stable states is dependent on the Oct-2+Grg/TLE levels, as can be seen from the positions of the minima in Figure 3. The model has a latency III level of EBNA-1 around 34,000 proteins (this value is calibrated to published data, see Methods) when no Oct-2+Grg/TLE complexes are present. This level decreases to around 14,000 after which point this latency state vanishes.

The EBNA-1 level in latency I varies less with Oct-2+Grg/TLE levels, remaining around 700–900 proteins (all values given for parameters presented in Table 1).

**Table 1:** Parameters used in model of the EBV genetic switch

**Name**	**Value**	**Reference**
*K*_dE_*	1 *nM *(0.1 – 10 *nM*)	Assumed model parameter
*K*_*dEFR*_	15 pM	[39]
*K*_dOFR_*	2.5 *nM *(2.5 – 12.5 *nM*)	[41]
*K*_*dQ*_	0.21 *μ*M	[39]
*E*_*tot *_in lat III	34,000 molecules	[43]
*τ*_1/2_	48 h	[44,45]
cell division time	24 h	[46]
Nucleus volume *	2*e*^-13^*l*(2*e*^-14 ^- 2*e*^-12^*l*)	[48]

Each Paramater is presented with a name, also used in the Methods section, and its value and corresponding reference puplication. *The three parameters that were altered in robustness tests. The range is given in Parenthesis.

### Promoter activity

**Figure 4:**
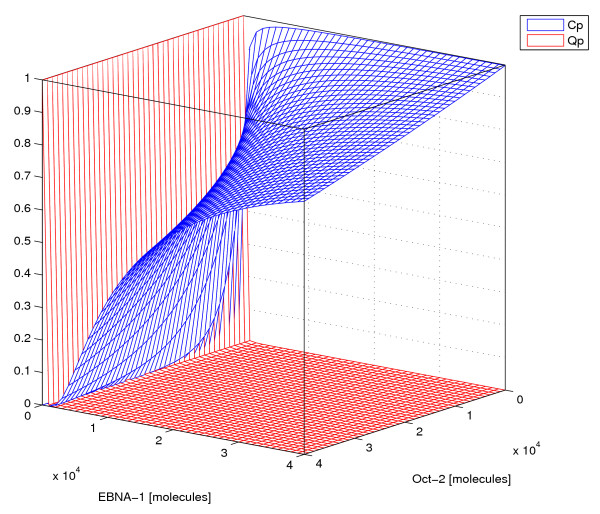
**Promoter activity**. The probability of transcription from both promoters, Cp (blue) and Qp (red) as function of EBNA-1 and Oct-2 proteins in the system. This plot shows the case when Oct-2+Grg/TLE has the maximum binding affinity to FR, *K*_*dOFR *_= 2.5 *nM*. The Qp activity is remarkably low for most EBNA-1 levels compared with the Cp activity.

Figure 4 shows computed Cp and Qp activity in our model, as functions of EBNA-1 and Oct-2+Grg/TLE levels, demonstrating the remarkably different activity levels of the two promoters. The Cp activity (blue surface) is 60–100% of maximum for all Oct-2+Grg/TLE and EBNA-1 levels shown. The Qp activity (red surface) is mostly around 1%, except at very low EBNA-1 levels. Since the half-life of EBNA-1 is long (see Methods), we expect Qp to only be active at a low level in latency I. Our model indeed shows very low Qp activity in latency I, both in the mono and bistable regions. It estimates the Qp activity to be about 1% of its full capacity in latency I, and close to zero in latency III. Cp on the contrary is active at 40–100% of maximum transcription rate in latency III, with the lower activity in the bistable region. In monostable latency I, the Cp activity is essentially zero and below 1% active for latency I in the bistable region. The Cp transcription is strongly dependent on Oct-2+Grg/TLE levels. However, at the system volumes tested here, Oct-2+Grg/TLE levels do not greatly affect Qp activity. To summarize, the two promoters are mutually exclusive in the monostable regions, although their activity levels differ, and also for the latency III state in the bistable region. In the latency I state in the bistable region, the Cp and Qp activities are somewhat comparable. Table 2 summarizes the Cp and Qp transcription activities presented above.

**Table 2:** Computed C and Q promoter activities for the mono- and bi-stable states in both latencies.

	Lat III		Lat I	
	bi	mono	bi	mono

Qp	-	-	≈1%	≈1%
Cp	40 – 90%	≥90%	≤1%	-

### Stability of the latency programs

**Figure 5:**
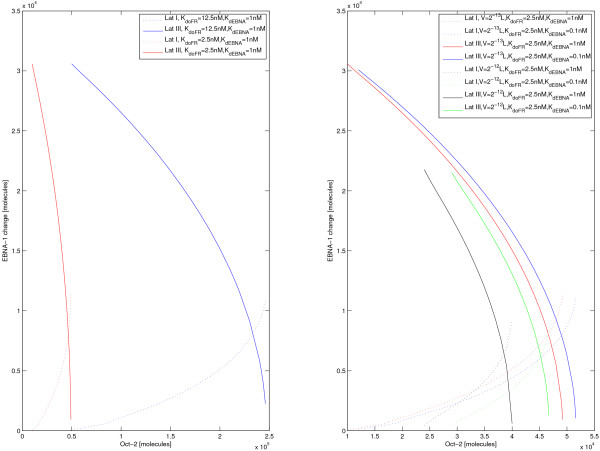
**Latency escapes and model robustness**. Figure showing the minimum instantaneous change in EBNA-1 protein number necessary to switch from latency I to III and vice versa, as a function of the number of Oct-2+Grg/TLR proteins. For small numbers only the latency III state exists, while for large numbers only latency I state exists, compare Figure 3 and Figure 4. Left figure: red lines are at the reference parameter values, in particular Oct-2 complex binding with affinity *K*_*dOFR *_= 2.5 *nM *and an EBNA-1 dimerization dissociation constant of 1 nM. Blue lines show a five-fold weaker Oct-2 affinity (*K*_*dOFR *_= 12.5 *nM*). Similar behaviour is then displayed at approximately five-fold higher Oct-2 level. Right figure: red solid and dotted lines at reference parameter values. Blue solid and dotted lines at a tenfold stronger EBNA-1 dimerization, and green solid and dotted lines at a tenfold greater volume. The latency III state is relatively robust towards either of these changes, while the latency I state changes more. Black solid and dotted lines show both a tenfold stronger EBNA-1 dimerization and a tenfold greater volume. This influences the latency III state more, essentially because EBNA-1 concentration in the latency III state is then comparable to the EBNA-1 dimerization dissociation constant.

The system volume was in our study estimated to be 2 * 10^-13 ^l (see Methods), but was increased and decreased ten-fold in sensitivity tests. For each volume size, the stable latency I and III levels of EBNA-1 was computed for different dimerization dissociation constants for EBNA-1; 10^-8 ^M, 10^-9 ^M and 10^-10 ^M, and varying levels of Oct-2. Stable steady state levels were also computed for a five-fold lower Oct-2 affinity to FR. The stability of the two latency states and their robustness to parameter changes can be quantified by the externally imposed change on EBNA-1 levels that induces the system to transit from one state to the other. This measure is of course appropriate only in the bi-stable region, where both states exist. As shown in Figure 5, for most parameters latency III is more stable than latency I, i.e., a larger change in EBNA-1 levels is needed to induce a transition from latency III to latency I than in the opposite direction. [see Additional file 1]

As the left plot in Figure 5 demonstrates, the model is certainly sensitive to changes in affinity of Oct-2 complex to FR. With a fivefold increase in affinity of Oct-2 the position of the bistable region is shifted towards five times higher Oct-2 levels, but the relative properties of the latency states do not change significantly. The right plot in Figure 5 illustrates the sensitivity of the model to changes in volume and dimerization constant for EBNA-1. The impact of changes in the dimerization constant depends on the system volume, where the larger volume is less robust. Comparing the red and blue lines in Figure 5, the effect of increasing the dimerization 10-fold results in a shift of the bistable region towards higher Oct-2 levels. This shift is substantially larger for the same dimerization parameter increase in the larger volume (black and green lines). The model's response to changes in the volume parameter is primarily that the bistable region shrinks with increasing volume, and latency I EBNA-1 levels rise.

### Inducing transitions

**Figure 6:**
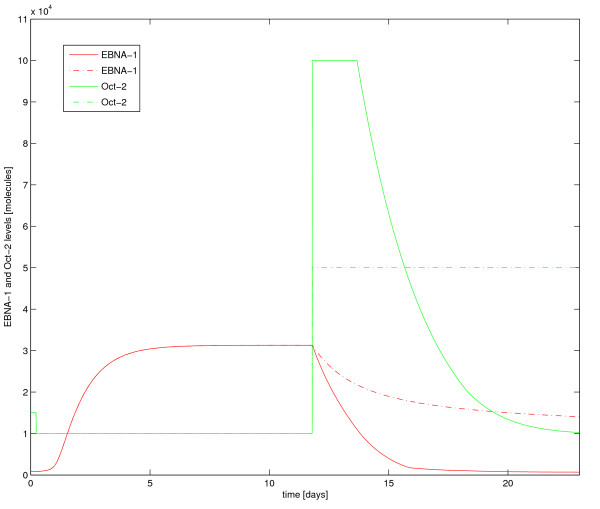
**Dynamics of EBNA-1 and Oct-2**. This plot illustrates how changes in Oct-2 levels affect the EBNA-1 levels. At time zero the system is stable in latency I, with an EBNA-1 level of 850 molecules, and an Oct-2 level of 15000 molecules. Transition to latency III is induced by lowering the Oct-2 level to 10000, activating the C promoter. Reaching the stable latency III level of EBNA-1 proteins thereafter take a few days. Induced switching back to resting latency I state demands a distinct increase in Oct-2, minimum a 10 fold change (green solid line). The greater increase in Oct-2 molecules the faster the cell is switched back to a stable latency I level of EBNA-1. The green solid and dashed line illustrate two different scenarios of elevated Oct-2 levels, where the red solid and dashed line are the corresponding resulting EBNA-1 levels.

In Figure 6 we illustrate how our model behaves when responding to changes in Oct-2+Grg/TLE. Initially, the cell is in latency I, with 850 EBNA-1 proteins molecules per cell and 15000 Oct-2+Grg/TLE complexes per cell. Decreasing the level of the Oct-2+Grg/TLE complex by 30% quickly leads to an increase in Cp activity and hence an increase in EBNA-1 production. Within four to five days EBNA-1 levels have reached the levels of stable latency III. Transitions from latency III to latency I on the other hand, requires quite high levels of the Oct-2+Grg/TLE complex for an extended period of time. The affinity of Oct-2 to FR being about 300 times weaker than the affinity of EBNA-1, Oct-2+Grg/TLE needs to be in large excess over EBNA-1 in order to fairly compete for binding to FR. Given a level of about 34,000 EBNA-1 in latency III, and the non-linear dependence of Cp activity on EBNA-1 concentration, one needs an Oct-2+Grg/TLE level of about 100,000 molecules, at least transiently, to lower EBNA-1 levels sufficiently in 5 days (solid lines in Figure 6). However, the switch can proceed slower with a lower level of Oct-2 for a longer time (dashed lines in Figure 6). Both transitions have their lower limits in time, due to maximum production rate and the slow degradation rate of EBNA-1.

## Discussion

EBV regulates fundamental properties of infected cells, and survives for long times in the host using different latency states, where we study the switch between the two extremes resulting in resting and proliferative states [1,3]. The molecular cell biology of the infection and EBVs association with various cancer types has been extensively studied. However, in order to fully understand how the virus survives in, and uses, its host cells we believe it is necessary to incorporate all information into a full, system level description. Our model of the switch between latency III and I is a first step towards such a model. The aim, besides opening the EBV research field to modeling, was to investigate whether EBNA-1 and Oct-2 regulation of Cp and Qp is sufficient to explain experimental results, and to identify important questions for further experimental studies.

A key effect in our model is the competitive binding of EBNA-1 and Oct-2+Grg/TLE to the Cp enhancer FR. Essential parameter values are hence binding affinities of EBNA-1 and Oct-2 to cognate sites. These binding affinities together determine the behaviour of the two involved promoters, and thereby also the switch between latency programs. Although it is well established that EBV can activate resting B-cells into proliferating blasts, the question how precisely this process is induced is still open. In our model, in order to keep the cell resting, using only Qp to produce EBNA-1, a high enough level of Oct-2+Grg/TLE is necessary in the cell. When Oct-2+Grg/TLE levels drop, EBNA-1 can access FR and trigger Cp transcription. Since Cp is positively auto-regulated, working on a high production rate, EBNA-1 levels then quickly increase once Cp has been turned on.

We show that our model of the EBV latency switch displays expected features of the switch. At different Oct-2 concentrations, the switch is either monostable, and then exhibits states of either latency I or latency III, or is bistable. This behavior is robust to several parameter changes, although the boundaries and size of the stability domains vary. That the bistable region extends up to a higher level of Oct-2 in the system, when lowering Oct-2 affinity to FR, is to be expected, since a lower affinity requires a higher Oct-2 level to inhibit Cp transcription. Also, the fact that the model's sensitivity to changes in EBNA-1 dimerization is correlated with the system volume, can be explained. With a smaller system volume, the concentration of EBNA-1 is higher. With a concentration of EBNA-1 higher than, or of same order as, the dimerization dissociation constant, there will be no significant impact in changing this parameter. Besides correlating with dimerization of EBNA-1, a noticeable effect of volume increase is the shrinkage of the bistable region, with respect to Oct-2 levels. This correlates with the larger number of EBNA-1 for latency I. Latency III amounts of EBNA-1 does not differ much between different parameter sets since it is determined from the production rate based on the measured number of EBNA-1 molecules (see Methods). The latency I levels however, depends only on the concentration of EBNA-1 in the cell, due to the negative feedback-loop, leading this number to vary.

Regarding promoter activities, there is experimental data for C and Q promoter transcripts in latency I and latency III cells [6,7]. Shaefer *et al*. present a Qp/Cp activity ratio of 5–100 in latency I cells, and 0.05–0.1 for latency III cells [6]. Zetterberg *et al*. show that latency I cells have 76–83% of all EBNA-1 transcripts originating from Qp, while less than 1% of EBNA-1 transcripts in latency III cells come from Qp [7]. Our theoretical predictions of activities of Cp and Qp presented in Table 2 correspond quite well to these experimental results. The mutual exclusiveness discussed in experimental papers agrees with our results, except for latency I in the bistable region. What is striking is the dramatic difference in predicted activity for the two promoters, even when comparing their respective stable latency states. Since the model is a simplified version of the *in vivo *mechanisms, our activities corresponds to the scenario without methylation or chromatin remodeling. This might explain the relatively low Qp/Cp activity ratio we see for latency I in the bistable region, since it is experimentally known that Cp is hypermethylated in latency I cells [34]. Lieberman's group recently published data showing that Cp can be regulated by changes in chromatin structure exerted by chromatin organizing proteins CTCF, which has one binding site in the EBV genome between FR and Cp [35]. This is compatible with our model as Grg/TLE can act as a chromatin remodelling protein by de-acetylating histones [36]. We believe it likely that the transcription factor recruitment and regulation is upstream of the chromatin regulating events in the switch, i.e. creates the signal that leads to chromatin remodelling.

There is as yet no experimental system to directly operate this switch in B cells in vitro. However there are models in EBV positive cell lines, which at least inefficiently mimics this switch, particularly the latency III to latency I transition by CD 40 ligand exposure or by RNA interference [37,38]. Experimental results indicate that proliferating EBV-transformed immunoblasts can be switched into a more restricted latency program within 5 days, due to a decrease in Cp activity [37]. According to our model, for this transition to occur in 5 days, Oct-2 levels need to be elevated up to 10-fold. The higher the Oct-2 level already in latency III, the lower fold increase of Oct-2 is necessary. The increase is however only required temporarily, since stable latency I state can persist even for quite low Oct-2 levels. Although a qualitative comparison between our experimental and theoretical molecular levels is possible, it is at this stage not enough to determine whether the switch is carried out between monostable states or in the bistable region. Further experiments with silencing of Oct-2 RNA followed by time series measurements on EBNA-1 and Oct-2 levels, should shed light on this matter.

Noise plays an important role in gene regulation and in models of gene regulation when the levels of regulatory factors are low, and in primarily dynamic phenomena, when there is a choice between different pathways. Noise also decreases stability and robustness of steady states. For most parameter values in the bistable region, the latency I state is more easily perturbed than latency III. A consequence is that a switch from latency I to latency III can be expected to occur in response to smaller external stimuli.

However, since there is no compelling reason for the EBV switch to operate in the bistable region, the influence of noise is likely to be small. Furthermore, the protein levels are relatively high in both latency states, reducing the impact of stochastic effects.

Although the structure of the FR invites consideration of cooperative binding of EBNA-1 (and/or Oct-2+Grg/TLE) to these binding sites, no experimental data is at present available. Stress testing the model involving these additional effects is in progress, and will be reported elsewhere. The addition of cooperative binding would not affect the qualitative functions of the switch, while it might influence the quantitative measurements, such as protein levels and the switching times. Another issue in modelling gene regulatory systems is whether important entire elements are left out. While this cannot be determined within a model, the propensity that it is the case can be adjudged from the model properties. We argue that our model is consistent with the knowledge present today, while it is clear that regulation of Oct-2 or Grg/TLE would logically complete the present model, as would information about other genetic feedback loops between the virus and its host cell. This then defines important tasks for the future.

## Conclusion

We have here shown that fundamental properties of the EBV infection, with high relevance for the virus as a risk factor in human cancer, can be modelled and understood mathematically. The model involves mechanisms which have, to our knowledge, only been used in models of simpler genetic systems before. However, also for this more complex system, these mechanisms nicely describe the dynamics and the properties of the viral genetic control.

A first conclusion is that EBNA-1 and Oct-2+Grg/TLE regulation of Cp and Qp is enough to establish mutually exclusive expression patterns, correlating with experimental data. Secondly, the switch manifests both mono- and bistable behavior, with the latency III state being markedly more robust. This in turn results in very different transition time scales between the latency states.

Our results and predictions moreover stress the importance, of the largely unknown regulation of the human transcription factor Oct-2 to the life-cycle of EBV.

## Methods

### Model base and main assumptions

As described in Background, our model is a simplification of the *in vivo *viral system, in the sense that it only involves promoter control by two transcriptional factors, EBNA-1 and Oct-2, with co-factors. The premise is that other regulatory factors documented in the literature, are less important as concerns the basic properties of the switch.

Regarding DNA binding of transcription factors to DNA, particularly to the FR region, the model assumes independent binding of both EBNA-1 and Oct-2. Moreover, bound proteins exclude only other bindings at that specific site, but do not affect neighbouring sites The octamer sites found in FR suggests a monomer binding of Oct-2, which we assume in our model, although the possibility of dimer bindings cannot be ruled out. The system is modelled with one kinetic equation, describing the dynamics of EBNA-1, coupled with thermodynamic equilibrium probabilities for transcription factor binding, to estimate transcription rates from Cp and Qp. As no regulation of Oct-2 levels by viral factors is known, Oct-2 level are essentially an external parameter to the model. The dynamics is modelled deterministically, disregarding noise in the transcription and translation (see Discussion). Details concerning parameter estimates are discussed in the following section.

### Parameters

It is evident that all modelling results depend on the parameters used, resulting in a demand for exact parameters, or good estimates based on experimental studies. In Table 1 we list the parameters used in this study. These include the EBNA-1 dimerization constant, DNA binding dissociation constants, steady state level of EBNA-1 in latency III together with EBNA-1 half-life time, cell division time and the volume of the system.

Of central importance in this model are the binding affinities for our transcription factors to the FR region and Qp sites. The dissociation constant for EBNA-1 binding to FR and the relative dissociation constant for the Qp sites was determined by Ambinder *et al*., 1990. Their experimental results give a dissociation constant, *K*_*dEFR*_, for EBNA-1 from FR that is 15 pM and a *K*_*dQ *_from the Qp binding sites that is 14 times higher [39]. Regarding EBNA-1 dimerization, EBNA-1 is known to be in dimer form in solution and bind DNA as a dimer [40], but the exact dimerization constant has however not been experimentally determined. We have used a *K*_*dE *_value of 1 nM as reference value, but varied this parameter to examine its impact on the system.

Oct-2 belongs to the POU family of proteins, which share two distinct DNA-binding subdomains; a POU-specific domain and a POU homeodomain. The ATGC half of the octamer sites is recognized by the POU-specific domain, and the homeodomain binds to the AAAT half [41]. Oct proteins therefore bind octamer sites as monomers, although they are able to form dimers and bind to similar palindromic sites known as MORE and PORE. In the FR region, the binding sites present are octamer-like; hence Oct-2 is assumed to bind as a monomer. The Oct-2 dissociation constant for the consensus octamer site, ATGCAAAT is known to be 2.5 nM [41]. The octamer sites in FR are however not perfect, and most sites noticeably have an A6G variant in the last half of the sequence, and at least two base exchanges in the first half. From published mutational analyses of Oct-2 and Oct-1 octamer sites we can however deduce that the homeodomain generally has a higher binding affinity that the POU-specific domain, and that an A6G mutation does not dramatically affect the affinity [41,42]. The dissociation constant of the Oct-2+Grg/TLE complex to sites within FR, *K*_*dOFR*_, is therefore here assumed to be 2.5 M but the effects of a higher dissociation constant was also tested.

The transcription rates for Cp and Qp and the translation rate for EBNA-1 transcripts are not experimentally known. As our model does not describe the transcription and translation steps individually but uses a total production rate, we incorporate both rates into one. Estimation of this production rate comes from experimentally determined steady state levels of EBNA-1 in latency III cell lines. Sternås *et al*. measured these EBNA-1 levels to range from 25,000 to 44,000 with an approximate mean value around 34,000 [43]. This roughly corresponds to a production of 34,000 new EBNA-1 proteins every cell cycle, when production is balanced by dilution and degradation of EBNA-1.

The exact half-life of EBNA-1, *τ*_1/2_, is not known, but experiments indicate that it is at least 48 hours, probably due to the Gly-Ala repeat domain which inhibits proteasomal degradation [44,45]. In our study we have hence chosen a half-life of 48 hours, and the cell division time is set to be 24 hours [46]. The model assumes an homogeneous distribution of molecules in the system and does not include spatial movements between different cellular compartments. EBNA-1 is mostly located in the nucleus, due to its functions in transcriptional and translational control [47]. Our system volume therefore corresponds to the nuclear volume of B-cells, a spherical volume with a diameter of 7 *μ*m [48].

### Physico-chemical model

The EBNA-1 dynamics is studied with a non-linear differential equation, (Eq. 1) describing production of EBNA-1 (E) from both promoters, Cp and Qp, protein degradation and also dilution, in the case of proliferating cells. The production from Cp is computed as the probability of gene transcription *P*_*c*_, times the rate of protein production *r*_*c *_(including transcription and translation), and likewise for Qp. Dilution is computed with a continuous dilution rate *r*_*dil*_, for cells that are in proliferating state. Degradation is computed with the continuous degradation rate *r*_*deg*_. The switch from resting to proliferating state is modelled instantaneously, meaning that the dilution rate is turned on as soon as Cp activity is higher than Qp activity, although in the real cell there is probably some delay. The total number of EBNA-1 molecules, *E*_*tot*_, in the system includes the free monomers, E, the dimers, *E*_*d*_, and the dimers bound specifically to DNA, *E*_*DNA*_, (Eq. 2). *E*_*DNA *_is computed as the mean value of bound EBNA-1 dimers and has to be calculated iteratively from the binding probabilities at each time step of a dynamic simulation, since it is dependent on the dimer concentration. The kinetic equation for EBNA-1 then reads;

dEtotdt=−Etot∗(rdeg+rdil)+rcPc+rqPq
 MathType@MTEF@5@5@+=feaafiart1ev1aaatCvAUfKttLearuWrP9MDH5MBPbIqV92AaeXatLxBI9gBamXvP5wqSXMqHnxAJn0BKvguHDwzZbqegyvzYrwyUfgarqqtubsr4rNCHbGeaGqiA8vkIkVAFgIELiFeLkFeLk=iY=Hhbbf9v8qqaqFr0xc9pk0xbba9q8WqFfeaY=biLkVcLq=JHqVepeea0=as0db9vqpepesP0xe9Fve9Fve9GapdbaqaaeGacaGaaiaabeqaamqadiabaaGcbaWaaSaaaeaacqWGKbazcqWGfbqrdaWgaaWcbaGaemiDaqNaem4Ba8MaemiDaqhabeaaaOqaaiabdsgaKjabdsha0baacqGH9aqpcqGHsislcqWGfbqrdaWgaaWcbaGaemiDaqNaem4Ba8MaemiDaqhabeaakiabgEHiQiabcIcaOiabdkhaYnaaBaaaleaacqWGKbazcqWGLbqzcqWGNbWzaeqaaOGaey4kaSIaemOCai3aaSbaaSqaaiabdsgaKjabdMgaPjabdYgaSbqabaGccqGGPaqkcqGHRaWkcqWGYbGCdaWgaaWcbaGaem4yamgabeaakiabdcfaqnaaBaaaleaacqWGJbWyaeqaaOGaey4kaSIaemOCai3aaSbaaSqaaiabdghaXbqabaGccqWGqbaudaWgaaWcbaGaemyCaehabeaaaaa@6A28@

where *E*_*tot *_is defined as;

*E*_*tot *_= 2*E*_*d *_+ *E *+ 2*E*_*DNA*_

### Transcriptional probability

The promoter transcription probability is computed from the probability of having a combination of transcription factors bound at the operator that allows for transcriptional activation. Full activation of Cp requires at least eight bound EBNA-1 dimers out of the 20 available sites at FR [14,15]. The independent binding of EBNA-1 and Oct-2+Grg/TLE means treating the 40 separate sites as 20, where each site can be occupied by either EBNA-1 or Oct-2+Grg/TLE. The transcription activity of Qp is dependent only on the EBNA-1 level, where transcription is blocked when one or two EBNA-1 dimers are bound.

In thermodynamic models, the probability of a certain combination of bound complexes is evaluated from the Boltzmann weight, *Z*, describing that state normalized with the sum of the weights for all possible states, *Z*_*tot*_. For FR, the statistical weight of having *n *EBNA-1 and *k *Oct-2+Grg/TLE bound to the *N *number of sites, *Z*_*nk*_, depend on the binding free energies of EBNA-1 and Oct-2+Grg/TLE, *E*_*eFR *_and *E*_*o*_, to their specific DNA sites, the concentrations of EBNA-1 and Oct-2+Grg/TLE, [*E*] and [*O*], and the number of different possible binding combinations that state can occur (Eq. 3). In the case of statistical weights for Q promoter activity, these are computed in the same manner, only dependent on EBNA-1 free energies, *E*_*eQ*_, and concentration [*E*] (Eq. 4). The number of binding sites, *N*, is 20 for FR and 2 for Qp.

Znk([E][O])=N!(N−n−k)!n!k![E]nenEeFR/kBT[O]kekOoFR/kBT
 MathType@MTEF@5@5@+=feaafiart1ev1aaatCvAUfKttLearuWrP9MDH5MBPbIqV92AaeXatLxBI9gBaebbnrfifHhDYfgasaacH8akY=wiFfYdH8Gipec8Eeeu0xXdbba9frFj0=OqFfea0dXdd9vqai=hGuQ8kuc9pgc9s8qqaq=dirpe0xb9q8qiLsFr0=vr0=vr0dc8meaabaqaciaacaGaaeqabaqabeGadaaakeaacqWGAbGwdaWgaaWcbaGaemOBa4Maem4AaSgabeaakiabcIcaOiabcUfaBjabdweafjabc2faDjabcUfaBjabd+eapjabc2faDjabcMcaPiabg2da9maalaaabaGaemOta4KaeiyiaecabaGaeiikaGIaemOta4KaeyOeI0IaemOBa4MaeyOeI0Iaem4AaSMaeiykaKIaeiyiaeIaemOBa4MaeiyiaeIaem4AaSMaeiyiaecaaiabcUfaBjabdweafjabc2faDnaaCaaaleqabaGaemOBa4gaaOGaemyzau2aaWbaaSqabeaacqWGUbGBcqWGfbqrdaWgaaadbaGaemyzauMaemOrayKaemOuaifabeaaliabc+caViabdUgaRnaaBaaameaacqWGcbGqaeqaaSGaemivaqfaaOGaei4waSLaem4ta8Kaeiyxa01aaWbaaSqabeaacqWGRbWAaaGccqWGLbqzdaahaaWcbeqaaiabdUgaRjabd+eapnaaBaaameaacqWGVbWBcqWGgbGrcqWGsbGuaeqaaSGaei4la8Iaem4AaS2aaSbaaWqaaiabdkeacbqabaWccqWGubavaaaaaa@6D03@

Zn([E])=N!n!(N−n)![E]nenEeFR/kBT
 MathType@MTEF@5@5@+=feaafiart1ev1aaatCvAUfKttLearuWrP9MDH5MBPbIqV92AaeXatLxBI9gBaebbnrfifHhDYfgasaacH8akY=wiFfYdH8Gipec8Eeeu0xXdbba9frFj0=OqFfea0dXdd9vqai=hGuQ8kuc9pgc9s8qqaq=dirpe0xb9q8qiLsFr0=vr0=vr0dc8meaabaqaciaacaGaaeqabaqabeGadaaakeaacqWGAbGwdaWgaaWcbaGaemOBa4gabeaakiabcIcaOiabcUfaBjabdweafjabc2faDjabcMcaPiabg2da9maalaaabaGaemOta4KaeiyiaecabaGaemOBa4MaeiyiaeIaeiikaGIaemOta4KaeyOeI0IaemOBa4MaeiykaKIaeiyiaecaaiabcUfaBjabdweafjabc2faDnaaCaaaleqabaGaemOBa4gaaOGaemyzau2aaWbaaSqabeaacqWGUbGBcqWGfbqrdaWgaaadbaGaemyzauMaemOrayKaemOuaifabeaaliabc+caViabdUgaRnaaBaaameaacqWGcbGqaeqaaSGaemivaqfaaaaa@519F@

### Experiments

#### Western Blot

Protein concentration of nuclear extracts of latency I and III cells was determined by Bio-Rad Dc protein assay (Bio-Rad, Hercules, CA). The same amount of nuclear extract was loaded on every lane. Proteins were fractionated by 9% sodium dodecyl sulfate-polyacrylamide gel electrophoresis (SDS-PAGE) and transferred to nitrocellulose membranes. After blocking for 1 h at room temperature with 5% milk, made up in PBS- 0.1% Tween 20 (PBST), the membranes were probed overnight at 4°C with the following antibodies at indicated dilutions. Anti Beta-Actin (Sigma) was used as second protein control; Oct-2 (Santa Cruz Biotechnologies, Santa Cruz, Calif.) were used at 1:2000 and anti- EBNA1 used at 1:1000 (OTX-1, a kind gift from Jaap Middeldorp, Amsterdam Free university). The second antibody used was horseradish peroxidase-conjugated (HRP) and bound immunocomplexes were detected by enhanced chemiluminscence (ECL; Amersham Life Science, Little Chalfont, Buckinghamshire, United Kingdom).

#### Chromatin immunoprecipitation assay (ChIP)

2 * 10^6 ^EBV latency type I Rael cell or latency III CBMI-Ral-Sto cell were collected. Proteins were cross-linked to DNA by adding formaldehyde to 1% of culture medium. Cell pellets were collected after 10 min incubation at 37°C. The cell pellets were resuspended in SDS lysis buffer, DNA sheared to 500–1000 bp by sonicating the lysate. The sonicated samples were diluted 10 fold and 2 ug of immunoprecipitating antibody was added respectively: OTiX, mouse monoclonal antibody anti EBNA1, (kindly provided by Dr Jaap Middeldorp, Amsterdam), anti-OCT2 (C-20); rabbit polyclonal antibody, Santa cruz Biotechnology, California; Goat polyclonal antibody Santa cruz Biotechnology, California, and rotated at 4C overnight. 100 ul of salmon sperm DNA/protein A agarose -50% slurry was added to collect antibody/protein/DNA complex. After washes and elution, the complexes were reverse crosslinked by adding sodium chloride to final concentration 0.2 M and kept at 65C for at least 6 hrs. Proteins were digested by proteinase K then DNA was purified by phenol/Chlorofome/ethanol precipitation. PCR primers were FRjz1S: TCCCTCTGGGAGAAGGGTAT and FRjz1A:TTTTCGCTGCTTGTCCTTTT for family of repeats (FR). For control experiments to exclude non-specific binding of antibodies to DNA, the immunopreciptated DNA was analyzed with primers to beta-actin: AC-S:

ATCATGTTTGAGACCTTCAA and AC-A: CATCTCTTGCTCGAAGTCCA. DNA from latency III cell lysate (Mutu III) was immunopreciptated with 4 ug of anti-Oct2 or 4 ug of anti-EBNA1 and amplified by PCR with primers to beta-actin. Water and reaction without antibody was used as negative controls, while cell lysate DNA prior to immunopreciptation was used as positive control.

## Authors' contributions

IE and EA designed the project. MW, IE and EA wrote the paper. MW and EA constructed the physico-chemical model of the EBV latency I/III switch. MW wrote the computer code and performed the simulations. JZ and JA performed the experiments reported in Figure 2. All authors read and approved the final manuscript.

## Supplementary Material

Additional file 1 Latency I and III levels of EBNA-1. This pdf-file includes tables with computed stable latency I and III levels of EBNA-1 for the different parameter sets tested. Click here for file

## References

[B1] Young LS, Rickinson AB (2004). Epstein-Barr virus: 40 years on. Nat Rev Cancer.

[B2] Almqvist J (2005). Epstein-Barr virus nuclear antigen 1, Oct & Groucho/TLE in control of promoter regulation. PhD thesis, Karolinska Institutet.

[B3] Amon W, Farrell PJ (2005). Reactivation of Epstein-Barr virus from latency. Reviews in Medical Virology.

[B4] Leight ER, Sugden B (2000). EBNA-1: a protein pivotal to latent infection bt Epsein-barr virus. Reviews in Medical Virology.

[B5] Schaefer BC, Woisetschlager M, Strominger JL, Speck SH (1991). Exclusive expression of Epstein-Barr virus nuclear antigen 1 in Burkitts lymphoma arises from a third promoter, distinct from the promoters used in latently infected lymphocytes. Proc Natl Acad Sci.

[B6] Schaefer BC, Strominger JL, Speck SH (1997). Host-cell-determined methylation of specific Epstein-Barr virus promoter regulates the choice between distinct viral latency programs. Molecular and Cellular Biology.

[B7] Zetterberg H, Stenglein M, Jansson A, Ricksten A, Rymo L (1999). Relative levels of EBNA1 gene transcripts from the C/W, F and Q promoters in Epstein-Barr virus-transformed lymphoid cells in latent and lytic stages of infection. Journal of General Virology.

[B8] Tsai CN, Liu ST, Chang YS (1995). Identification of a novel promoter located within the BamHIQ region of the Epstein-barr virus genome for the EBNA 1 gene. DNA and Cell Biology.

[B9] Nonkwelo C, Ruf IK, Sample J (1997). The Epstein-Barr virus EBNA-1 promoter Qp requires an intitator-like element. Journal of Virology.

[B10] Bodescot M, Perricaudet M, Farrell PJ (1987). A promoter for the highly spliced EBNA family of RNAs of Epstein-Barr virus. Journal of Virology.

[B11] Almqvist J, Zou J, Linderson Y, Borestrom C, Altiok E, Zetterberg H, Rymo L, Petterson S, Ernberg I (2005). Functional interaction of Oct transcription factors with the family of repeats in Epstein-Barr virus *ori*P. Journal of General Virology.

[B12] Reisman D, Sugden B (1986). Trans activation of an Epstein-Barr viral transcriptional enhancer by the Epstein-Barr viral nuclear antigen 1. Molecular and Cellular Biology.

[B13] Sugden B, Warren N (1999). A promoter of Epstein-Barr virus that can function during latent infection can be transactivated by EBNA-1, a viral protein required for viral DNA repliction during latent infection. Journal of Virology.

[B14] Wysokenski DA, Yates JL (1989). Multiple EBNA1-binding sites are required to form an EBNA1-dependent enhancer and to activate a minimal replicative origin within *oriP *of Epstein-Barr virus. Journal of Virology.

[B15] Zetterberg H, Borestrom C, Nilsson T, Rymo L (2004). Multiple EBNA1-binding sites within oriPI are required for EBNA1-dependent transactivation of the Epstein-Barr virus C promoter. International Journal of Oncolocy.

[B16] Malin S, Linderson Y, Almqvist J, Ernberg I, Tallone T, Petterson S (2005). DNA-dependent conversion of Oct-1 and Oct-2 into transcriptional repressors by Groucho/TLE. Nucleic Acids Research.

[B17] Sung NS, Kenney S, Gutch D, Pagano JS (1991). EBNA-2 transcativates a lymphoid-specific enhancer in the *Bam*HI C promoter of Epstein-Barr virus. Journal of Virology.

[B18] Ling PD, Rawlins DR, Hayward SD (1993). The Epstein-Barr virus immortalizing protein EBNA-2 is targeted to DNA by a cellular enhancer-binding protein. Proc Natl Acad Sci.

[B19] Kupfer SR, Summers WC (1990). Identification of a Glucocorticoid-responsive element in Epstein-Barr virus. Journal of Virology.

[B20] Nilsson T, Zetterberg H, Wang YC, Rymo L (2001). Promoter-proximal regulatory elements involved in *oriP*-EBNA1-independent and -dependent activation of the Epstein-Barr virus C promoter in B-lymphoid cell lines. Journal of Virology.

[B21] Puglielli MT, Woisetschlaeger M, Speck SH (1996). oriP is essential for EBNA gene promoter activity in Epstein-Barr virus-immortalized lymphoblastoid cell lines. Journal of Virology.

[B22] Roder K, Wolf S, Larkin K, Schweizer M (1999). Interaction between the two ubiquitously expressed transcription factors NF-Y and Sp1. Gene.

[B23] Guerra RF, Imperadori L, Mantovani R, Dunlap DD, Finzi L (2007). DNA compaction by the nuclear factor Y (NF-Y). Biophys J.

[B24] Mantovani R (1999). Review: The molecular biology of the CCAAt-binding factor NF-y. gene.

[B25] Schaefer BC, Strominger JL, Speck SH (1995). Redefining the Epstein-Barr virus-encoded nuclear antigen EBNA-1 gene promoter and transcription initiation site in group I Burkitt lymphoma cell lines. Proc Natl Acad Sci.

[B26] Schaefer BC, Paulson E, Strominger JL, Speck SH (1997). Constitutive activation of Epstein-Barr virus (EBV) nuclear antigen 1 gene transcription by IRF1 and IRF2 during restricted EBV latency. Molecular and Cellular Biology.

[B27] Sung NS, Wilson J, Davenport M, Sista ND, Pagano JS (1994). Reciprocal regulation of the Epstein-Barr virus *Bam*HI-F promoter by EBNA-1 and an E2F transcription factor. Molecular and Cellular Biology.

[B28] Ruf IK, Sample J (1999). Repression of Epstein-Barr virus EBNA-1 gene transcription by pRb during restricted latency. Journal of Virology.

[B29] Sample J, Henson EB, Sample C (1992). The Epstein-Barr virus nuclear protein 1 promoter active in type I latency is autoregulated. J Virol.

[B30] Shea M, Ackers G (1985). The OR control system of bacteriophage lambda. A physical-chemical model for gene regulation. J Mol Biol.

[B31] Bintu L, Buchler NE, Garcia HG, Gerland U, Hwa T, Kondev J, Kuhlman T, Phillips R (2005). Transcriptional regulation by the numbers: models. Curr Opin Genet Dev.

[B32] Reinitz J, Vaisnys JR (1990). Theoretical and experimental analysis of the phage lambda genetic switch implies missing levels of co-operativity. Journal of Theoretical Biology.

[B33] Aurell E, Brown S, Johanson J, Sneppen K (2002). Stability puzzles in phage lambda. Physical Review E.

[B34] Salamon D, Takacs M, Ujvari D, Uhlig J, Wolf H, Minarovits J, Niller HH (2001). Protein-DNA binding and CpG methylation at nucleotide resolution of latency-associated promoters Qp, Cp, and LMP1p pf Epstein-Barr virus. J Virol.

[B35] Chau CM, Zhang XY, McMahon SB, Lieberman PM (2006). Regulation of Epstein-Barr virus latency type by the chromatin boundary factor CTCF. J Virol.

[B36] Brantjes H, Roose J, van De Wetering M, Clevers H (2001). All Tcf HMG box transcription factors interact with Groucho-related co-repressors. Nucleic Acids Res.

[B37] Pokrovskaja K, Ehlin-Henriksson B, Kiss C, Challa A, Gordon J, Gogolak P, Klein G, Szekely L (2002). CD40 ligation downregulates EBNA-2 and LMP-1 expression in EBV-transformed lymphoblastoid cell lines. Int J Cancer.

[B38] Hong M, Murai Y, Kutsuna T, Takahashi H, Nomoto K, ans Shin Ishizawa CMC, Zhao QL, Ogawa R, Harmon BV, Tsuneyama K, Takano Y (2006). Suppression of Epstein-barr nuclear antigen 1 (EBNA1) by RNA interference inhibits proliferation of EBV-positive Burkitt's lymphoma cells. Journal of Cancer Research and Clinical Oncology.

[B39] Ambinder RF, Shah WA, Rawlins DR, Hayward GS, Hayward SD (1990). Definition of the sequence requirements for binding of the EBNA-1 protein to its palindromic target sites in Epstein-Barr virus DNA. Journal of Virology.

[B40] Frappier L, O'Donnell M (1991). Overproduction, purification and characterization of EBNA1, the origin binding protein of Epstein-Barr virus. The Journal of Biological Chemistry.

[B41] Shah PC, Bertolino E, Singh H (1997). Using altered specificity Oct-1 and Oct-2 mutants to analyze the regulation of immunoglobulin gene transcription. The EMBO Journal.

[B42] Verrijzer C, Alkema M, van Weperen W, Leeuwen HV, Strating M, van der Vliet P (1992). The DNA binding specificity of the bipartite POU domain and its subdomains. EMBO J.

[B43] Sternas L, Middleton T, Sugden B (1990). The average number of molecules of Epstein-Barr nuclear antigen 1 per cell does not correlate with the average number of Epstein-Barr virus (EBV) DNA molecules per cell among different clones of EBV-immortalized cells. J Virol.

[B44] Davenport MG, Pagano JS (1999). Expression of EBNA-1 mRNA is regulated by cell cycle during Epstein-Barr virus type I latency. Journal of Virology.

[B45] Levitskaya J, Sharipo A, Leonchiks A, Ciechanover A, Masucci MG (1997). Inhibition of ubiquitin/proteasome-dependent protein degradation by hte Gly-Ala repeat domain of the Epstein-Barr virus nuclear antigen 1. Proc Natl Acad Sci USA.

[B46] Woo KB, Funkhouser WK, Sullivan C, Alabaster O (1980). Analysis of the proliferation kinetics of Burkitt's lymphoma cells. Cell Tissue Kinteics.

[B47] Daikoku T, Kudoh A, Fujita M, Sugaya Y, Isomura H, Tsurumi T (2004). In vivo dynamics of EBNA-oriP interaction during latent and lytic replication of Epstein-Barr virus. Journal of Biological Chemistry.

[B48] Abbas AK, Lichtman AH, Pober JS (2000). Cellular and molecular immunology.

