# Supplementary material for: Epstein-Barr virus latency switch in human B-cells: a physico-chemical model
Source: BMC Syst Biol. 2007 Aug 31;1:40. doi: 10.1186/1752-0509-1-40 (PMC2164963; doi:10.1186/1752-0509-1-40)
Supplement: Additional file 1 — Latency I and III levels of EBNA-1. This pdf-file includes tables with computed stable latency I and III levels of EBNA-1 for the different parameter sets tested. [file 1752-0509-1-40-S1.pdf]

Latency I and III levels of EBNA-1

**Kd**	$1*10^{-8}$ **M**		$1*10^{-9}$ **M**		$1*10^{-10}$ **M**	
**Oct [molecules]**	Lat I	Lat III	Lat I	Lat III	Lat I	Lat III
-	-	33980	-	33980	-	33980
1000	-	33728	-	33735	-	33737
2000	-	33473	-	33488	-	33493
3000	-	33217	-	33239	-	33246
4000	-	32959	182.9	32989	164.03	32998
5000	216.97	32698	207.54	32736	187.05	32748
6000	212.23	32436	173.98	32482	162.57	32496
7000	210.45	32171	170.97	32225	158.52	32242
8000	209.32	31904	169.56	31967	156.88	31986
9000	208.42	31634	168.6	31706	155.86	31728
10000	207.66	31362	167.82	31443	155.06	31468
11000	206.98	31088	167.14	31178	154.39	31206
12000	206.37	30811	166.54	30910	153.79	30941
13000	205.81	30531	166	30640	153.25	30674
14000	205.31	30248	165.5	30367	152.76	30404
15000	204.84	29962	165.04	30092	152.3	30132
16000	204.42	29672	164.62	29813	151.89	29857
17000	204.02	29380	164.23	29532	151.5	29579
18000	203.66	29084	163.86	29248	151.14	29298
19000	203.32	28785	163.53	28960	150.8	29014
20000	203	28481	163.21	28669	150.49	28727
21000	202.71	28174	162.91	28375	150.19	28437
22000	202.43	27863	162.64	28077	149.92	28143
23000	202.18	27547	162.38	27775	149.66	27845
24000	201.93	27226	162.13	27470	149.41	27544
25000	201.71	26901	161.9	27160	149.18	27239
26000	201.49	26570	161.68	26845	148.96	26929
27000	201.29	26234	161.48	26526	148.75	26615
28000	201.09	25892	161.28	26202	148.56	26296
29000	200.91	25543	161.1	25872	148.37	25972
30000	200.74	25188	160.92	25537	148.2	25642
31000	200.57	24825	160.75	25195	148.03	25307
32000	200.42	24454	160.59	24847	147.87	24966
33000	200.27	24075	160.44	24492	147.71	24618
34000	200.12	23686	160.29	24130	147.57	24263
35000	199.99	23287	160.15	23759	147.43	23901
36000	199.86	22877	160.02	23380	147.29	23530
37000	199.73	22453	159.89	22990	147.16	23150
38000	199.62	22016	159.77	22590	147.04	22760
39000	199.5	21562	159.65	22178	146.92	22359
40000	199.39	21090	159.54	21753	146.81	21946
41000	199.29	20596	159.43	21312	146.7	21520
42000	199.19	20077	159.33	20854	146.59	21078
43000	199.09	19527	159.23	20377	146.49	20618
44000	199	18939	159.13	19876	146.4	20139
45000	198.9	18302	159.04	19347	146.3	19635
46000	198.82	17599	158.95	18785	146.21	19102
47000	198.73	16798	158.86	18179	146.12	18534
48000	198.65	15831	158.78	17516	146.04	17921
49000	198.58	14464	158.69	16772	145.96	17247
50000	198.5	-	158.62	15900	145.88	16485
51000	198.43	-	158.54	14773	145.8	15581
52000	198.36	-	158.47	-	145.73	14367
53000	198.29	-	158.4	-	145.66	-
54000	198.22	-	158.33	-	145.59	-
55000	198.16	-	158.26	-	145.52	-
56000	198.1	-	158.2	-	145.45	-
57000	198.04	-	158.13	-	145.39	-
58000	197.98	-	158.07	-	145.33	-
59000	197.92	-	158.01	-	145.27	-
60000	197.86	-	157.95	-	145.21	-

Table 1: EBNA-1 levels in the two stable latency states for system volume $V = 2 * 10^{-14}$ L, an Oct-2 dissociation constant to FR of 2.5 nM and three different dimerization dissociation constants for EBNA-1.

**Kd**	$1 * 10^{-8}$ **M**		$1 * 10^{-9}$ **M**		$1 * 10^{-10}$ **M**	
**Oct [molecules]**	Lat I	Lat III	Lat I	Lat III	Lat I	Lat III
0	-	33905	-	33912	-	33914
1000	-	33627	-	33659	-	33668
2000	-	33347	-	33404	-	33421
3000	-	33065	-	33147	-	33172
4000	-	32780	-	32888	-	32921
5000	-	32492	-	32628	-	32668
6000	-	32201	-	32364	-	32413
7000	-	31907	-	32099	-	32156
8000	934.6	31610	-	31831	-	31897
9000	987.55	31309	-	31561	-	31635
10000	1038.4	31005	673.45	31288	-	31372
11000	1087.4	30697	710.45	31013	-	31106
12000	1134.8	30386	746.49	30735	623.69	30837
13000	1180.8	30070	781.68	30454	655.45	30566
14000	1225.6	29751	816.11	30170	686.61	30293
15000	1072.5	29426	849.85	29884	717.2	30016
16000	1048	29098	882.98	29594	747.3	29737
17000	1035.6	28764	915.53	29300	776.94	29455
18000	1028.3	28425	823.02	29003	806.14	29170
19000	1023.6	28081	791.8	28703	834.96	28882
20000	1020.5	27730	777.61	28399	719.02	28590
21000	1018.3	27374	769.27	28090	699.45	28295
22000	1016.7	27010	763.87	27778	688.85	27996
23000	1015.5	26640	760.17	27461	682.18	27693
24000	1014.6	26261	757.53	27139	677.68	27386
25000	1013.9	25874	755.59	26812	674.49	27075
26000	1013.3	25478	754.13	26480	672.16	26759
27000	1012.8	25072	753.01	26142	670.41	26439
28000	1012.4	24656	752.13	25798	669.07	26113
29000	1012	24227	751.42	25448	668.02	25782
30000	1011.7	23784	750.84	25090	667.19	25445
31000	1011.4	23326	750.36	24726	666.51	25102
32000	1011.1	22852	749.94	24353	665.95	24752
33000	1010.9	22357	749.59	23971	665.48	24395
34000	1010.7	21840	749.27	23579	665.08	24031
35000	1010.4	21296	748.99	23177	664.73	23658
36000	1010.2	20719	748.73	22763	664.42	23275
37000	1010	20102	748.49	22336	664.15	22883
38000	1009.8	19433	748.27	21895	663.9	22480
39000	1009.6	18693	748.06	21436	663.67	22064
40000	1009.4	17849	747.87	20959	663.45	21635
41000	1009.3	16826	747.68	20458	663.26	21190
42000	1009.1	15370	747.5	19932	663.07	20727
43000	1008.9	-	747.33	19372	662.89	20244
44000	1008.8	-	747.17	18773	662.72	19736
45000	1008.6	-	747.01	18120	662.56	19199
46000	1008.4	-	746.86	17394	662.4	18626
47000	1008.3	-	746.71	16558	662.25	18006
48000	1008.1	-	746.56	15522	662.11	17324
49000	1008	-	746.42	13881	661.96	16552
50000	1007.9	-	746.28	-	661.83	15631
51000	1007.7	-	746.14	-	661.69	14377
52000	1007.6	-	746.01	-	661.56	-
53000	1007.5	-	745.88	-	661.43	-
54000	1007.3	-	745.76	-	661.31	-
55000	1007.2	-	745.63	-	661.19	-
56000	1007.1	-	745.51	-	661.07	-
57000	1007	-	745.39	-	660.95	-
58000	1006.8	-	745.28	-	660.84	-
59000	1006.7	-	745.16	-	660.72	-
60000	1006.6	-	745.05	-	660.61	-

Table 2: EBNA-1 levels in the two stable latency states for system volume $V = 2 * 10^{-13}$ L, an Oct-2 dissociation constant to FR of 2.5 nM and three different dimerization dissociation constants for EBNA-1.

**Kd**	$1*10^{-8}$ **M**		$1*10^{-9}$ **M**		$1*10^{-10}$ **M**	
**Oct [molecules]**	Lat I	Lat III	Lat I	Lat III	Lat I	Lat III
0	-	32878	-	33157	-	33230
1000	-	32492	-	32873	-	32972
2000	-	32099	-	32586	-	32712
3000	-	31699	-	32296	-	32450
4000	-	31291	-	32004	-	32185
5000	-	30875	-	31708	-	31919
6000	-	30450	-	31409	-	31649
7000	-	30016	-	31106	-	31378
8000	-	29571	-	30800	-	31103
9000	-	29115	-	30490	-	30826
10000	-	28647	-	30176	-	30547
11000	-	28165	-	29858	-	30264
12000	-	27668	-	29536	-	29979
13000	-	27155	-	29209	-	29690
14000	-	26622	-	28877	-	29398
15000	4510.3	26068	-	28540	-	29103
16000	4613.4	25489	-	28198	-	28804
17000	4713.9	24881	-	27851	-	28501
18000	4812.2	24238	-	27497	-	28194
19000	4908.3	23553	-	27136	-	27883
20000	5002.5	22815	-	26769	-	27568
21000	5094.8	22006	-	26394	-	27248
22000	5185.4	21099	-	26011	-	26924
23000	5274.3	20040	-	25619	-	26594
24000	5361.8	18694	3034.4	25217	-	26258
25000	5447.8	16208	3092.9	24805	-	25917
26000	5532.5	-	3150.6	24382	-	25570
27000	5615.9	-	3207.6	23945	-	25215
28000	5698	-	3264	23495	-	24854
29000	5779.1	-	3319.7	23028	2541.9	24484
30000	5859	-	3374.7	22543	2589.1	24107
31000	5937.9	-	3429.2	22037	2635.9	23720
32000	6015.7	-	3483.1	21506	2682.2	23322
33000	6092.7	-	3536.5	20945	2728.2	22914
34000	6168.7	-	3589.4	20348	2773.7	22493
35000	6243.8	-	3641.7	19706	2818.9	22058
36000	5705.4	-	3693.6	19003	2863.7	21608
37000	5562.6	-	3745	18216	2908.2	21139
38000	5472.2	-	3796	17297	952.3	20650
39000	5407.2	-	3846.6	16124	2996.1	20136
40000	5357.4	-	3896.7	13672	3039.5	19593
41000	5317.9	-	3946.5	-	3082.7	19014
42000	5285.7	-	3995.9	-	3125.6	18388
43000	5259.1	-	4044.9	-	3168.2	17702
44000	5236.7	-	4093.5	-	3210.5	16927
45000	5217.7	-	4141.8	-	3252.6	16009
46000	5201.5	-	4189.7	-	3294.4	14790
47000	5187.5	-	4237.4	-	3335.9	-
48000	5175.3	-	4284.7	-	3377.2	-
49000	5164.7	-	4331.7	-	3418.3	-
50000	5155.5	-	4074.7	-	3459.2	-
51000	5147.3	-	3926.8	-	3499.8	-
52000	5140.1	-	3843.5	-	3540.2	-
53000	5133.7	-	3785	-	3580.3	-
54000	5128.1	-	3740.5	-	3620.3	-
55000	5123.1	-	3705.1	-	3660.1	-
56000	5118.6	-	3676	-	3699.6	-
57000	5114.6	-	3651.6	-	3670.6	-
58000	5111	-	3630.9	-	3422.9	-
59000	5107.8	-	3613.1	-	3329.3	-
60000	5104.9	-	3597.6	-	3267.3	-

Table 3: EBNA-1 levels in the two stable latency states for system volume $V = 2*10^{-12}$ L, an Oct-2 dissociation constant to FR of 2.5 nM and three different dimerization dissociation constants for EBNA-1.

**Kd**	$1 * 10^{-8}$ **M**		$1 * 10^{-9}$ **M**		$1 * 10^{-10}$ **M**	
**Oct [molecules]**	Lat I	Lat III	Lat I	Lat III	Lat I	Lat III
0	-	33905	-	33912	-	33914
1000	-	33850	-	33862	-	33865
5000	-	33628	-	33660	-	33669
10000	-	33349	-	33406	-	33423
15000	-	33068	-	33150	-	33174
20000	-	32784	-	32892	-	32924
25000	-	32497	-	32632	-	32672
30000	-	32207	-	32370	-	32418
31000	-	32149	-	32317	-	32367
32000	-	32090	-	32264	-	32316
33000	-	32031	-	32211	-	32265
34000	-	31973	-	32158	-	32213
35000	-	31914	-	32105	-	32162
36000	-	31855	-	32052	-	32110
37000	-	31796	-	31999	-	32059
38000	911.29	31737	-	31946	-	32007
39000	922.25	31677	-	31892	-	31956
40000	933.1	31618	-	31839	-	31904
45000	985.93	31318	-	31569	-	31643
50000	1036.6	31016	672.15	31298	-	31381
55000	1085.5	30709	9.06	31024	590	31116
60000	1132.9	30399	745.01	30747	622.36	30848
65000	1178.8	30085	780.12	30467	654.04	30579
70000	1223.5	29766	814.46	30184	685.11	30306
75000	1074.5	29443	848.12	29899	715.63	30031
80000	1048.9	29116	881.16	29610	745.65	29753
85000	1036.2	28784	913.63	29318	775.2	29472
90000	1028.7	28446	826.56	29022	804.34	29188
95000	1023.9	28103	793.09	28723	833.08	28901
1e+05	1020.7	27755	778.35	28420	721.06	28610
1.05e+05	1018.4	27400	769.76	28113	700.46	28316
1.1e+05	1016.8	27038	764.21	27802	689.48	28019
1.15e+05	1015.6	26669	760.42	27486	682.62	27717
1.2e+05	1014.7	26293	757.72	27166	677.99	27412
1.25e+05	1013.9	25908	755.74	26840	674.72	27102
1.3e+05	1013.3	25514	754.25	26510	672.34	26788
1.35e+05	1012.9	25111	753.11	26174	670.55	26469
1.4e+05	1012.4	24696	752.21	25832	669.18	26145
1.5e+05	1011.7	23831	750.9	25128	667.26	25480
1.6e+05	1011.2	22905	749.99	24394	666.01	24791
1.7e+05	1010.7	21902	749.31	23626	665.12	24074
1.8e+05	1010.2	20793	748.76	22816	664.46	23324
1.9e+05	1009.8	19524	748.3	21954	663.93	22534
2e+05	1009.5	17974	747.89	21026	663.48	21695
2.1e+05	1009.1	15639	747.53	20010	663.1	20796
2.2e+05	1008.8	-	747.19	18867	662.75	19815
2.3e+05	1008.5	-	746.88	17516	662.43	18720
2.4e+05	1008.2	-	746.58	15716	662.13	17443
2.5e+05	1007.9	-	746.3	-	661.85	15806
2.6e+05	1007.6	-	746.04	-	661.58	-
2.7e+05	1007.4	-	745.78	-	661.33	-
2.8e+05	1007.1	-	745.54	-	661.09	-
2.9e+05	1006.9	-	745.3	-	660.86	-
3e+05	1006.6	-	745.07	-	660.64	-
3.5e+05	1005.6	-	744.07	-	659.64	-
4e+05	1004.8	-	743.22	-	658.8	-
4.5e+05	1004.1	-	742.5	-	658.08	-
5e+05	1003.4	-	741.87	-	657.46	-
5.5e+05	1002.9	-	741.33	-	656.92	-
6e+05	1002.4	-	740.85	-	656.44	-

Table 4: EBNA-1 levels in the two stable latency states for system volume $V = 2 * 10^{-13}$ L, an Oct-2 dissociation constant to FR of 12.5 nM and three different dimerization dissociation constants for EBNA-1.
